# Development and validation of monoclonal antibodies specific for *Candida albicans* Als2, Als9-1, and Als9-2

**DOI:** 10.1371/journal.pone.0269681

**Published:** 2022-07-08

**Authors:** Soon-Hwan Oh, David A. Coleman, Xiaomin Zhao, Lois L. Hoyer

**Affiliations:** Department of Pathobiology, University of Illinois at Urbana-Champaign, Urbana, IL, United States of America; Yonsei University, REPUBLIC OF KOREA

## Abstract

Fungal agglutinin-like sequence (Als) cell-surface glycoproteins, best characterized in *Candida albicans*, mediate adhesive and aggregative interactions with host cells, other microbes, and abiotic surfaces. Monoclonal antibodies (MAbs) specific for each *C*. *albicans* Als protein are valuable reagents for gaining insight into Als protein localization and function. This manuscript describes development and validation of MAbs specific for *C*. *albicans* Als2, as well as for *C*. *albicans* Als9-1 and Als9-2, two protein variants produced from the *ALS9* locus. Native *C*. *albicans ALS9* expression levels were not sufficiently high to produce detectable Als9 protein on the wild-type cell surface so MAb validation required production of overexpression strains, each featuring one of the two *ALS9* alleles. An anti-Als2 MAb was raised against an N-glycosylated form of the protein immunogen, as well as an Endoglycosidase H-treated immunogen. The MAb raised against the N-glycosylated immunogen proved superior and immunolabeled *C*. *albicans* yeast cells and germ tubes, and the surface of *Candida dubliniensis* and *Candida tropicalis* yeasts. Als2 was visible on *C*. *albicans* yeast cells recovered from a murine model of oral candidiasis, demonstrating Als2 production both *in vivo* and *in vitro*. These new MAbs add to the collection of anti-Als MAbs that are powerful tools to better understand the role of Als proteins in *C*. *albicans* biology and pathogenesis.

## Introduction

Agglutinin-like sequence (Als) proteins are large, cell-surface glycoproteins that function in adhesive and aggregative interactions [reviewed in 1]. *Candida albicans* has eight distinct *ALS* loci (*ALS1* to *ALS7*, *ALS9*); many species in the Saccharomycetales have at least one *ALS* gene [2]. Als proteins have a secretory signal peptide at the N-terminal end and a GPI anchor addition site at the C-terminal end that direct their processing and modification via the secretory pathway to a final localization linked to ß-1,6-glucan in the fungal cell wall [3]. The structure of the Als adhesive N-terminal (NT) domain was solved by x-ray crystallography and features a peptide-binding cavity that is required for ligand recognition [4, 5]. Als adhesive interactions involve host cells, other microbes, and protein-coated surfaces such as denture or catheter materials [1]. Als aggregative interactions are mediated by an amyloid-forming region that is part of the N-terminal domain [6]. Although the general NT-domain structure appears to be conserved among Als proteins across a wide variety of fungal species [2], sufficient sequence variation exists to use the NT domain to raise monoclonal antibodies (MAbs) that specifically recognize individual Als proteins.

Previous reports documented use of *Pichia pastoris*-produced Als NT protein immunogens to raise murine MAbs against several of the *C*. *albicans* Als proteins [7–10]. Here, we report development and validation of anti-Als MAbs that augment the collection. Protein immunogens included *C*. *albicans* Als2 [11, 12], as well as Als9-1 and Als9-2, two proteins that are 84% identical in the NT domain, produced from the *C*. *albicans ALS9* locus [7, 13, 14]. The *ALS9* alleles are widespread among the *C*. *albicans* clades [13]. Because the Als9-2 NT domain functions in adhesion to cultured human vascular endothelial cells but the Als9-1 NT domain does not [14], availability of specific anti-Als9-1 and anti-Als9-2 MAbs will benefit future analyses. This report also features initial use of the new MAbs to provide further insight into the dynamics of the *C*. *albicans* Als family.

## Materials and methods

### Monoclonal antibodies

Methods for raising anti-Als MAbs were published previously [7] and are reproduced briefly here for the reader’s convenience. CUG-codon-corrected *C*. *albicans* Als NT domain fragments were cloned into pPIC3 and transformed into *Pichia pastoris* GS115 (Invitrogen). Sequences from coordinates 1 to 984 of *ALS2* (GenBank accession number AF024582), *ALS9-1* (AY269423), and *ALS9-*2 (AY269422) were used to direct synthesis of hexa-His-tagged Als NT domain fragments (amino acids 18–328) that were secreted using their own signal sequence. Proteins were purified from culture supernatant using ammonium sulfate precipitation and His-Trap column chromatography (GE Healthcare). Proteins were dialyzed in phosphate-buffered saline and concentrations determined using the Micro BCA Protein Assay kit (Thermo Scientific). Endoglycosidase H (Endo H; Roche) was used according to manufacturer’s instructions to remove the N-linked carbohydrate.

Proteins were used to immunize BALB/c mice intraperitoneally [7]. Splenic lymphocytes were fused with murine myeloma tumor cells (SP2/0) and selected in hypoxanthine aminopterin thymidine medium. Enzyme-linked immunosorbent assays were used to screen for antibody production and for specificity of the antibody for the immunogen and none of the other Als proteins [7]. Western blotting was used for additional confirmation of MAb specificity [7]. The MAb raised against the glycosylated (non-Endo-H-treated) Als2 immunogen was named 2-C6. The anti-Als2 MAb raised against the deglycosylated Als2 immunogen was named 2-C7. Anti-Als9 immunogens were both Endo H-treated prior to immunizing the mice. Anti-Als9-1 was named 9-C4; anti-Als9-2 was named 9-A7. All MAbs described here were IgG1 with a kappa light chain as determined using the Monoclonal Antibody Isotyping Kit (Pierce).

### Biacore analysis of MAb-antigen binding affinity

A Biacore 3000 SPR instrument with version 4.1 control software was used for protein binding kinetics and affinity measurements. The CM5 sensor chip, amine coupling kit, rabbit anti-mouse Fc antibody (RamFc), glycine solutions (10 mM, pH 1.5 and pH 2.0), sodium acetate (10 mM, pH 5.0), 0.5 N NaOH, and 10% Surfactant P20 were purchased from GE Healthcare (Piscataway, NJ). HBS-EP running buffer (10 mM HEPES pH 7.4, 150 mM NaCl, 3 mM EDTA, 0.0005% Surfactant P20) was made in-house. Als protein fragments were made and purified as described by Coleman et al. [7].

The CM5 chip was docked in the Biacore 3000, normalized with 70% glycerol and cleaned twice with 0.5 N NaOH by injection at 20 μl/min flow rate for 30 sec before ligand surface preparation. RamFc was covalently immobilized to the CM5 surface by performing the standard amine coupling procedure, increasing surface response units (RU) by approximately 5,000 RU. Anti-Als MAbs (50 μg/ml) were then captured to the surfaces by manual injection at a flow rate of 5 μl/min until about 350 RU net increase was achieved. Control flow cells had no anti-Als MAb.

Als proteins were assayed for binding to their corresponding MAbs for kinetics and affinity measurements. For each binding-assay cycle, a protein of known concentration was injected to flow cells with or without MAb (control surface) simultaneously at a 20 μl/min flow rate with 2 min association and various lengths of dissociation time using the kinject command. After the dissociation phase, the reaction surface was regenerated to remove antibody-antigen complex from the sensor chip surface by injecting 10 mM glycine, pH 1.75 for 30 sec. The Als2 (Endo H-treated) protein was an exception because it dissociated from the MAb surface completely after 10 min dissociation thus no glycine regeneration was used and no MAb re-charge was needed between each binding cycle. The integrated fluidic cartridge (IFC) and injection needle were washed between assay cycles if glycine was used.

Analyte concentration series and dissociation time for each assay were: 35, 70, 140, 280, 560 and 1120 nM, and 5 min dissociation for the Als9-2 assay; 3.9, 7.8, 15.6, 31.3, 62.5 and 125 nM, and 5 min dissociation for Als9-1 assay; 0.2, 0.4, 0.8, 1.6, 3.2 and 6.4 μM, and 10 min dissociation for Als2 (Endo H-treated) assay; 16.9, 33.9, 67.5, 135, 271, 1843 nM, and 5 min dissociation for Als2 (glycosylated) assay. Running buffer instead of analyte was injected at the beginning, in the middle, and at the end of each analyte concentration series assay. The resulting buffer blanks were used to correct bulk shift and other instrument noise during data analysis. Each assay was repeated three times.

Biacore sensorgrams were analyzed using BIAevaluation software (version 4.1). Analyte binding curves subtracted from controls were trimmed and aligned by performing X- and Y-transformations. The curves were adjusted from blanks during Y-transformation. Curve fitting was conducted by using simultaneous k_d_ and k_a_ fitting with the 1:1 binding model with mass transfer. The resulting parameters from three replicates were used to produce the mean and standard deviation for each assay. Chi-square values were calculated for the average squared residual per data point to judge the closeness of fit of the data to the predicted model with Chi-square values ideally < 2.0.

### *ALS2* promoter deletion and reintegration constructs

Previous difficulty deleting the second *C*. *albicans ALS2* allele from the SC5314 strain background led to attempts to control *ALS2* expression by integrating inducible promoters in place of the remaining *ALS2* promoter in strain 1443 (*ALS2/als2Δ-ura3*) [12]. Integration of the *MAL2* promoter led to a strain with considerably decreased *ALS2* expression [12]. The tetracycline-regulatable (Tet) promoter described by Nakayama et al. [15] was also considered for use. Attempts to construct a tetracycline-regulatable *ALS2* allele were unsuccessful, but led to strain 2757, derived from strain 1443 [12]. Strain 2757 featured deletion of one *ALS2* allele and integration of a disruption cassette in place of the *ALS2* promoter upstream of the second *ALS2* allele. The strain had nearly unmeasurable *ALS2* transcription (see below). This section details construction of strain 2757 and its companion strain (2801) into which a functional *ALS2* promoter was restored to drive expression of one *ALS2* allele.

Restriction enzymes were purchased from Thermo Fisher Scientific or New England Biolabs and used according to manufacturer instructions. PCR screening of transformant candidates used *Taq* polymerase (Thermo Fisher Scientific). PCR amplifications for cloning used *Pfu* (proofreading) or *Pfu* Turbo polymerase (Stratagene). Agarose gel purification of PCR products and restriction fragments used 0.7% agarose/Tris Acetate-EDTA gels (40 mM Tris, 20 mM acetic acid, 1 mM EDTA, pH 8.0). Gel fragments were excised and DNA purified using the GeneClean III kit (MP Biomedicals). Ligations used T4 DNA Ligase (Thermo Fisher Scientific) according to manufacturer instructions. Ligations were transformed into *Escherichia coli* TOP10 competent cells (Thermo Fisher Scientific) and selectively plated onto Luria Broth agar (per liter: 10 g tryptone, 10 g sodium chloride, 5 g yeast extract with 15 g Bacto agar to solidify the medium) supplemented with the appropriate antibiotic, typically ampicillin (Sigma-Aldrich) at 100 μg/ml. Yeast transformations used a spheroplast method [16]; transformants were selected on plates of synthetic complete medium without uridine (SC-Uri) [17] containing 1 M sorbitol. Transformants were grown in SC-Uri liquid medium and genomic DNA extracted using the MasterPure Yeast DNA Purification kit (Epicentre). Southern blotting used the Genius system (Roche). Accuracy of constructs was checked by Sanger DNA sequencing at the Roy J. Carver Biotechnology Center, University of Illinois Urbana-Champaign.

The region upstream of *ALS2* was amplified from *C*. *albicans* strain SC5314 [18] genomic DNA with primers 2TR-AKpnIF and 2TR-AXhoIR (Table 1). The 1004-bp fragment, which included the 1003 bp upstream of *ALS2* and the A of the *ALS2* ATG start codon, was designed to delete 1003 nt upstream of *ALS2* following transformation of the construction cassette. PCR products were digested with restriction enzymes *Kpn*I and *Xho*I, then agarose-gel purified. The fragment was cloned into vector p99-CAU1 [15] that had been digested with the same enzymes. The resulting vector was called p99-CAU1-flankingA.

**Table 1:** Primer sequences used in *C*. *albicans* strain constructions.

Primer Name	Primer Sequence (5’– 3’)
2TR-AKpnIF	CCC GGT ACC TGA TTT ATC TAC GGA GAT AGC
2TR-AXhoIR	CCC CTC GAG AGT CTT GTC TGG TTT GGT TTG
2TR-BNotIF	CCC GCG GCC GCT TTC AAA TGC TTT TAC AAT TTT TG
2TR-BSstIR	CCC GAG CTC AAC CGT TAG AAT TAG CTT CAC
URA-XhoIF	TTT CTC GAG GTG AAT TGT AAT ACG ACT CAC
URA-SmaIR	TTT TCC CGG GAG GTC GAC GGT ATC GAT AAG C
ALS2upF	CCC CCT AGG GAT GTT TGC ATT ACG CTG TTG
ALS2upR	CCC CTC GAG CCA TAA GGT TGT TGA AAA CAT C
2pRC-HindF	CCC AAG CTT TGA TTT ATC TAC GGA GAT AGC
2pRC-SstIIF	CCC CCG CGG CAA ACC AAA CCA GAC AAG ACT CG
2pRC-SstIR	CCC GAG CTC CAG CAG TAG TAC TGT CAG TAA CTG
9LA-XhoF	CCC CTC GAG ATG CTA CCA CAA TTC CTA TTG
ALS9XbaIR	CCC TCT AGA TTA AAT AAA CAA AAA TAA TAT TGT GAC C
9SA-XhoF	CCC CTC GAG ATG CTT CCA CAA TTC ATA TTG
9BglR	CCC AGA TCT TTA AAT AAA CAA AAA TAA TAT TGT
RT-ALS9F	CAT CAT TTG TGT CTA CAA CTG CTG
RT-ALS9R	GAA CCC TTT GTT TCT GAA TAT GG

The 5’ end of the *ALS2* gene was amplified from SC5314 genomic DNA using primers 2TR-BNotIF and 2TR-BSstIR (Table 1). The 976-bp fragment included -6 to 970 of the *ALS2* coding region. PCR products were digested with *Not*I-*Sst*I, agarose-gel purified, then cloned into p99-CAU1-flankingA that had been similarly digested. The resulting plasmid, 2685 (p99-CAU1-2TR) encoded (in linear order) upstream *ALS2* sequences, *URA3*, the tetracycline-regulatable promoter, and the 5’ *ALS2* region.

The *URA3* gene in plasmid 2685 was replaced with a copy of *URA3* flanked by direct repeats so that the Ura marker could be recycled when integrating a wild-type copy of the *ALS2* promoter. The *URA3* fragment flanked by direct repeats (*URA3-dpl200*) was amplified by PCR from plasmid pDDB57 [19] using primers URA-XhoIF and URA-SmaIR (Table 1). PCR products were digested with *Xho*I and *Sma*I, agarose gel purified and GeneCleaned as described above. The fragment was cloned into *Xho*I-*Sma*I-digested plasmid 2685. The resulting plasmid was named 2717.

Plasmid 2717 was digested with restriction enzymes *Sma*I and *Not*I to remove the tetracycline promoter from the plasmid. After agarose gel purification, the fragment was treated with *Pfu* polymerase to create blunt ends. The blunt ends were religated and the vector transformed into *E*. *coli* TOP10. The resulting plasmid was named 2756 and contained the *ALS2* promoter deletion cassette. The cassette was released with *Kpn*I-*Sst*I digestion, agarose gel purified and GeneCleaned. Approximately 10 μg of purified cassette was transformed into *C*. *albicans* strain 1443 spheroplasts. Transformants were selected on SC-Uri plates with 1 M sorbitol.

Transformants were streaked on SC-Uri plates for purification and grown in liquid SC-Uri for genomic DNA extraction. Restriction-enzyme-digested genomic DNA was Southern blotted with the *ALS2* promoter fragment amplified using primers ALS2upF and ALS2upR (Table 1). A strain that was lacking the *ALS2* promoter and that had the same growth rate as the control strain (CAI12) [20] was identified and named 2757. Zhao et al. [16] described the method for growth rate analysis.

A different transformation cassette was constructed to reintegrate the wild-type *ALS2* promoter into strain 2757. An *ALS2* upstream flanking fragment was amplified from SC5314 genomic DNA using primers 2pRC-HindF and 2TR-AXhoIR (Table 1). PCR products were digested with *Hin*dIII and *Xho*I then agarose gel purified and GeneCleaned. The fragment was cloned into *Hin*dIII-*Xho*I-digested plasmid pUL [16]. An *ALS2* promoter and partial coding region fragment was amplified from SC5314 genomic DNA using primers 2pRC-SstIIF and 2pRC-SstIR (Table 1). The PCR product was digested with *Sst*II-*Sst*I, agarose gel purified and GeneCleaned, then cloned into the growing vector that was digested with the same enzymes. The resulting plasmid contained the *ALS2* promoter reintegration cassette that was released with *Hin*dIII-*Sst*I digestion.

*C*. *albicans* strain 2757 was plated on agar medium containing 5-fluoroorotic acid to select for excision of the *URA3* marker [21]. Resulting colonies were analyzed by Southern blot as described above. The ura^-^ strain was named 2796. The *ALS2* promoter reintegration cassette was transformed using the spheroplast method and transformants selected as described above. Strain 2801 was verified as correct using Southern blotting and demonstrated to have the same growth rate as CAI12 [16].

### Construction of *ALS9* overexpression strains

Information presented here details construction of strain 3299 in which *ALS9-1* expression is driven by the *TPI1* promoter; the construct was integrated into the *C*. *albicans ALS9* locus. Details are also provided for construction of strain 2965 in which *ALS9-2* expression is driven by the *TPI1* promoter with the construct integrated at the *RP10* locus. Overexpression strains were needed to produce sufficient cell-surface quantities for Als9-1 or Als9-2 to validate specificity of the anti-Als9 MAbs described here. General methods for PCR amplification, restriction enzyme digestion, transformation, and screening of transformants were presented above.

The *ALS9-1* overexpression construct was built in plasmid 1105 [22] which was derived from CIp10 [23]. *ALS9-1* was amplified from a cloned, sequence-verified copy in plasmid 2953. Amplification used primers 9LA-XhoF and ALS9XbaIR (Table 1). The PCR product was digested with *Xho*I-*Xba*I and cloned into similarly digested plasmid 1105. The *Xho*I site in plasmid 1105 was downstream of the *TPI1* promoter; the *Xba*I site was downstream of the *RP10* sequence. The resulting vector (3297) was linearized with *Bst*Z17I, which cut at nt 884 of the *ALS9-1* coding region. The linearized plasmid was transformed into *C*. *albicans* CAI4 [24]. The correct construct (3299) had *ALS9-1* expression driven by the *TPI1* promoter, terminated with the native *ALS9-1* termination signal, followed by *URA3*, the ampicillin resistance gene and ColEI origin from CIp10 integrated at the *ALS9-1* locus.

Plasmid 1499, which consisted of *ALS9-2* cloned into the pCRBlunt vector (Invitrogen), was used as the template for PCR amplification of *ALS9-2* with primers 9SA-XhoF and 9BglR (Table 1). The fragment was digested with *Xho*I-*Bgl*II, gel purified, and cloned into plasmid 1105 [22] that had been digested with the same enzymes. Transformants were screened by PCR with primers RT-ALS9F and RT-ALS9R (Table 1). The resulting plasmid was named 2920. Plasmid 2920 was linearized with *Nco*I which cut in the *RP10* sequence. Transformation of the linearized plasmid into *C*. *albicans* strain CAI4 directed the overexpression construct to the *RP10* locus. The correct transformant was identified by Southern blotting and named 2965.

### Immunolabeling *C*. *albicans* cells

Methods for immunolabeling *C*. *albicans* cells were described by Coleman et al. [7]. Endo H treatment of cells aided immunolabeling visualization of Als proteins. For Endo H treatment, *C*. *albicans* cells were recovered from culture by centrifugation and washed twice in DPBS. Cells were resuspended in 50 mM potassium acetate, pH 5.0 and 1 × proteinase inhibitor cocktail (Roche catalog number 11836153001) in a total volume of 0.3 ml. Endo H (0.05 unit; Roche catalog number 11088726001) was added and the tube placed in a 360-degree rotator for 5 h at 37°C. Following incubation, cells were washed twice with DPBS and fixed with 3% paraformaldehyde for 10 min. Fixed cells were washed twice with DPBS and stored in DPBS at 4°C until used for immunolabeling.

For some experiments, the *C*. *albicans* cell surface was covalently labeled with Alexa Fluor 594 according to the method of Coleman et al. [8]. Labeling of the inoculum cells prior to *in vitro* culture growth was used to distinguish them from cells of subsequent generations in the culture flask population.

### Murine model of oral candidiasis

The murine model of oral candidiasis, and recovery and preservation of *C*. *albicans* cells from the murine infection, were described by Coleman et al. [10]. Use of animals was reviewed and approved by the University of Illinois Institutional Animal Care and Use Committee under protocol number 11197. Mice were euthanized by pentobarbital sodium injection. All efforts were made to minimize animal suffering.

### Real-time RT-PCR assays

Real-time RT-PCR analysis was used to measure the effect of *ALS2* deletion on *ALS4* expression. Primers and a detailed method were reported in Zhao et al. [12]. Absolute quantification of transcript copy number was also described in that report.

### Bioinformatics resources

The *Candida* Gene Order Browser (cgob.ucd.ie) [25] was used to detect *C*. *albicans ALS* gene orthologs as identified by synteny (conserved chromosomal location) in other *Candida* species. Sequence alignments and calculation of percent identity used Clustal Omega (http://www.ebi.ac.uk/Tools/msa/clustalo) [26]. Oh et al. [2] lists the amino acid sequences for the protein immunogens used to raise the various MAbs, as well as the corresponding N-terminal domain sequences across the Als family in *C*. *dubliniensis*, *C*. *tropicalis*, and other closely related fungal species.

## Results

### Monoclonal antibody specificity and characteristics

**Fig 1:**
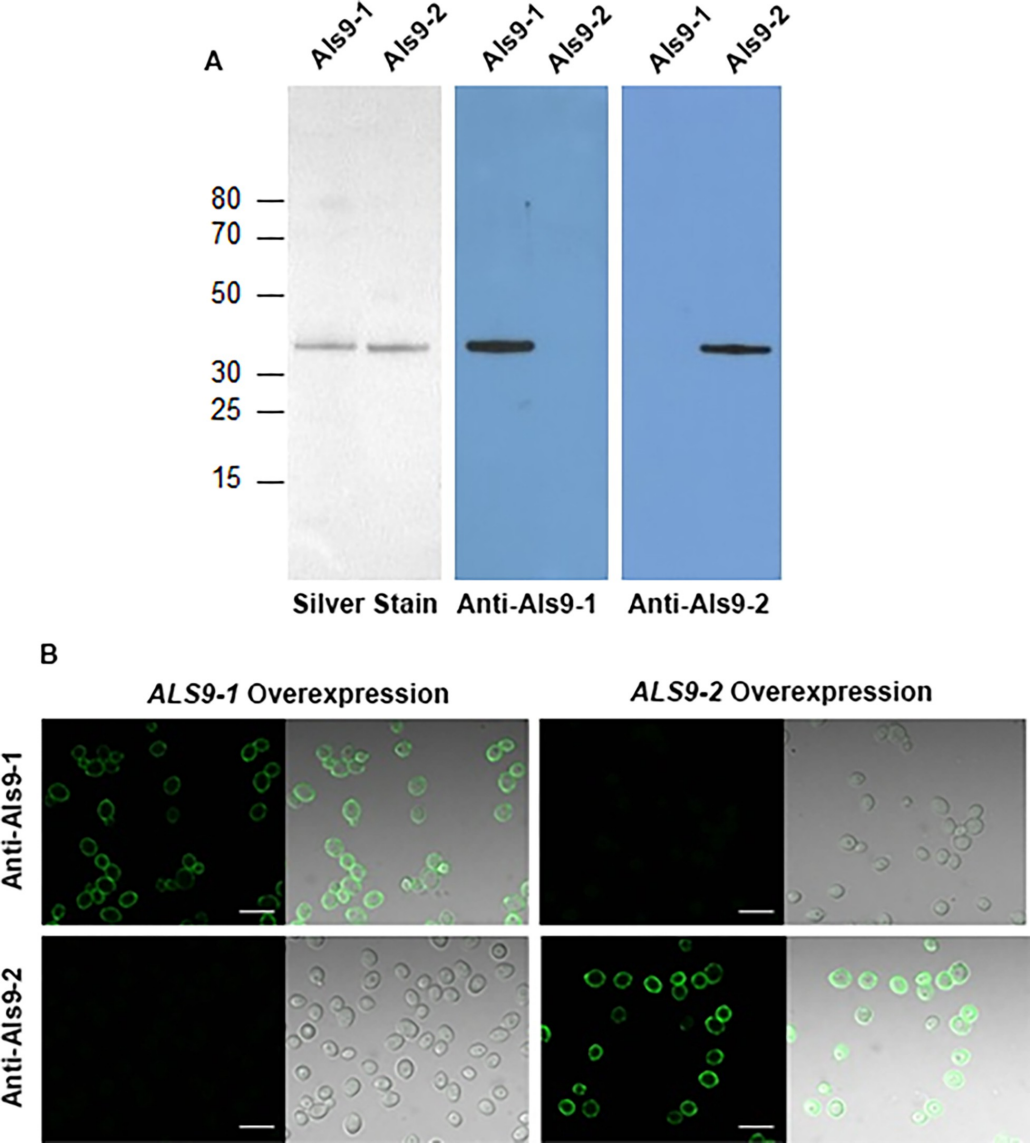
Specificity of anti-Als9-1 and anti-Als9-2 for their respective proteins. (A) Western blotting of purified NT domain fragments (amino acids 18 to 328 for each) from Als9-1 and Als9-2. The silver-stained polyacrylamide gel validated equal loading for the proteins (left panel). Western blots (middle and right panels) of identical sets of lanes were also prepared and exhibited specificity of each MAb for its antigen. (B) Immunolabeling of *ALS9* overexpression strains with anti-Als9-1 MAb 9-C4 or anti-Als9-2 MAb 9-A7. Immunolabeling did not produce visible signals for wild-type *C*. *albicans* so overexpression strains were constructed. Each produced protein under control of the constitutive *TPI1* promoter. Labeling only of cells that expressed the allele used to produce the antigen further validated specificity of the anti-Als9 MAbs. Endo H treatment of the *ALS9-2* overexpression cells was required to see a positive immunolabeling signal with MAb 9-A7. The *ALS9-1* overexpression strain did not require EndoH treatment to see its positive signal after immunolabeling with anti-Als9-1 MAb 9-C4. Scale bars in each figure = 10 μm.

Endo H-treated Als9-1 (amino acids 18–328) and Als9-2 (amino acids 18–328) were used as immunogens to raise specific mouse MAbs. Specificity of each MAb for its respective immunogen was demonstrated by Western blotting (Fig 1A). MAbs 9-C4 and 9-A7 were used to immunolabel the surface of wild-type *C*. *albicans* cells grown under various combinations of conditions of growth medium (YPD, RPMI 1640), temperature (30°C, 37°C), time (early, log-phase, saturated cultures), and Endo H treatment without success. However, surface labeling of *C*. *albicans* strains that overexpressed one of the two *ALS9* alleles was successful, further validating the specificity of MAb 9-C4 for Als9-1 and MAb 9-A7 for Als9-2 (Fig 1B). Detection of Als9-2 on the *C*. *albicans* overexpression strain surface required Endo H treatment of the cells prior to immunolabeling. Biacore measurement of antigen-antibody binding kinetics for anti-Als9-1 (9-C4) provided a dissociation constant (K_D_) of 32 ± 6 nM (Chi-square = 0.3 ± 0.2). The K_D_ value for anti-Als9-2 (9-A7) was 71 ± 14 nM (Chi-square = 1.1 ± 0.9).

**Fig 2:**
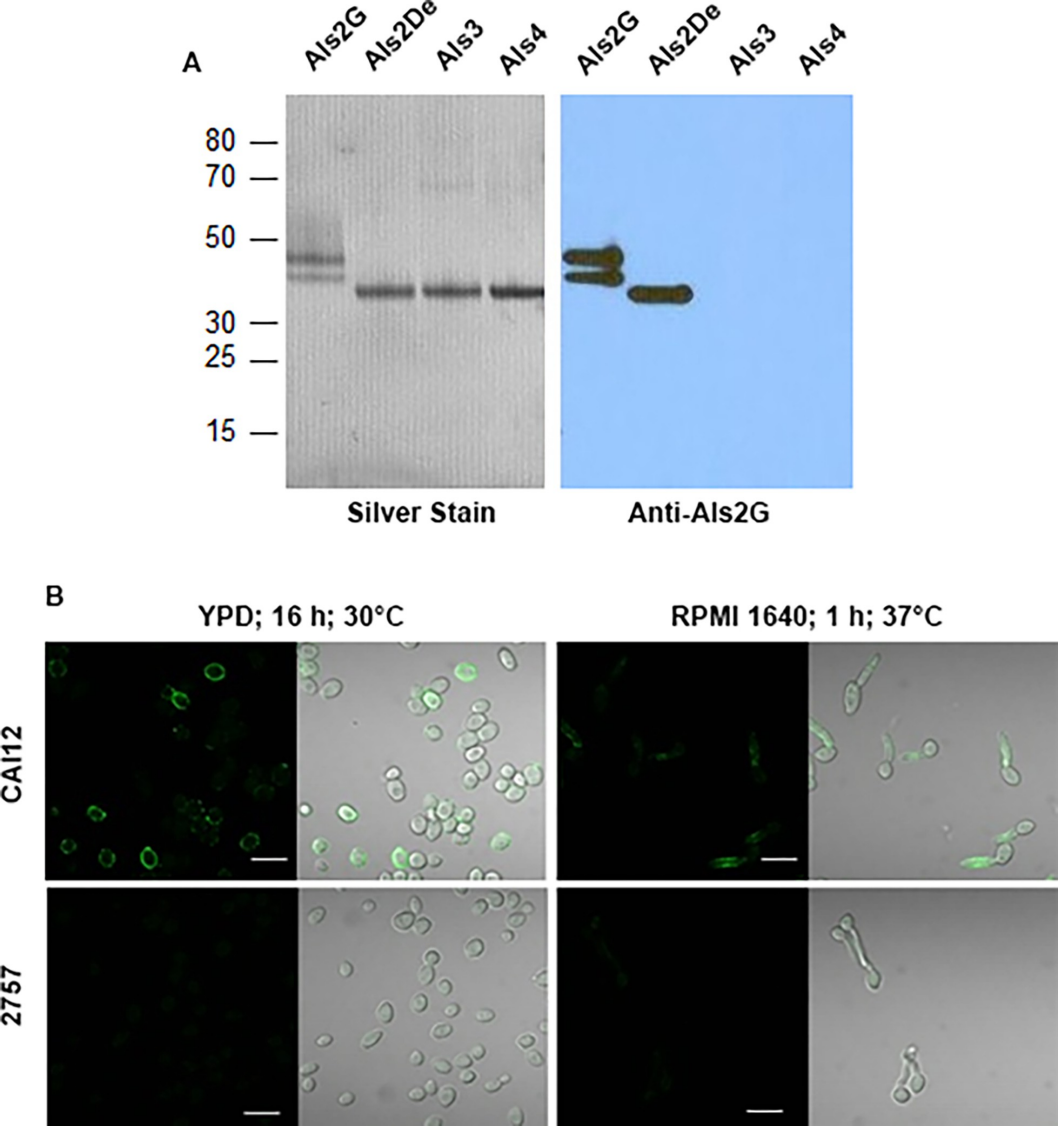
Western blotting and immunolabeling of *C*. *albicans* cells to demonstrate specificity of anti-Als2 MAb 2-C6 for Als2. (A) Purified N-terminal fragments of Als2 (amino acids 18–328) untreated (G) or treated (De) with EndoH, Als3 (amino acids 18–329), and Als4 (amino acids 18–329) were separated by polyacrylamide gel electrophoresis and silver stained (left panel; 0.5 μg of each protein). 250 ng of each protein were run in additional lanes of the polyacrylamide gel, transferred to a Hybond-P PVDF membrane (Amersham) and Western blotted with the anti-Als2 MAb 2-C6 (raised against the glycosylated form of the protein). MAb 2-C6 recognized both the glycosylated and Endo H-treated forms of Als2, but did not recognize Als3 or Als4, consistent with the conclusion that MAb 2-C6 was specific for Als2. (B) Additional evidence for its Als2 specificity was secured by using anti-Als2 MAb 2-C6 to immunolabel the surface of *ALS2/ALS2* strain CAI12 and strain 2757 which has near-zero *ALS2* expression. Immunolabeling only of the wild-type cell surface suggested specificity of the MAb. The immunolabeling pattern of yeast was punctate, and only visible on a fraction of the cells in a 16-h culture. Labeling of the germ tube surface for cells grown at 37°C for 1 h in RPMI medium was diffuse. Scale bars in each image indicate 10 μm.

An anti-Als2 MAb was raised against the glycosylated form of the immunogen. This MAb was named 2-C6 and had a K_D_ of 8.2 ± 0.8 nM (Chi-square = 1.1 ± 0.6). MAb 2-C6 recognized both the glycosylated (G) and Endo H-treated (De) forms of Als2 on a Western blot and did not react with other Als protein fragments, indicating its specificity for Als2 (Fig 2A). MAb 2-C6 immunolabeling was visible on the surface of *C*. *albicans ALS2/ALS2* yeast cells and germ tubes but was not detected on strain 2757 which produced very little *ALS2* transcript (Fig 2B). Reintegration of the wild-type *ALS2* promoter to restore *ALS2* transcription yielded strain 2801, for which anti-Als2 MAb 2-C6 labeling was not visible. Quantitative real-time PCR analysis of these strains was conducted using yeast cells grown in YPD for 16 h at 37°C. Means and standard deviations of *ALS2* transcript copy number from three data points on two separate days were 28615 ± 3510 for strain CAI12, 33 ± 11 for strain 2757, and 3130 ± 777 for strain 2801. Therefore, although *ALS2* transcription increased in strain 2801, reintegration of the *ALS2* promoter did not restore wild-type transcription levels and did not produce sufficient cell-surface protein for immunolabeling visualization.

Another attempt to raise a specific anti-Als2 MAb used the Endo H-treated form of the immunogen, resulting in MAb 2-C7. Binding kinetic analysis for MAb 2-C7 showed a K_D_ of 13 ± 0.8 nM (Chi-square = 0.17 ± 0.01). Although MAb 2-C7 recognized the surface of wild-type *C*. *albicans* cells, it also produced a slight background on strain 2757 indicating the presence of a cross-reactive epitope. Because MAb 2-C6 did not produce the background immunolabeling of strain 2757, it was considered the preferred MAb and used in subsequent studies.

*ALS4* transcription was a focus for analysis in the newly created *C*. *albicans* strains because previous work suggested that *ALS4* expression increased when *ALS2* expression was compromised [12]. Real-time RT-PCR analysis showed that *ALS4* was overexpressed by 1.9 ± 0.4-fold (mean and standard deviation of three separate experiments) in strain 2757 compared to CAI12 for cells grown for 16 h in YPD at 37°C.

### Anti-Als2 immunolabeling of *Candida* cells

Because wild-type *ALS9* transcription levels were too low to visualize Als9 cell-surface protein with the anti-Als9 MAbs, additional experimentation was not pursued. The ability to see anti-Als2 2-C6 immunolabeling prompted initial exploration of Als2 localization on *C*. *albicans* and other closely related species.

**Fig 3:**
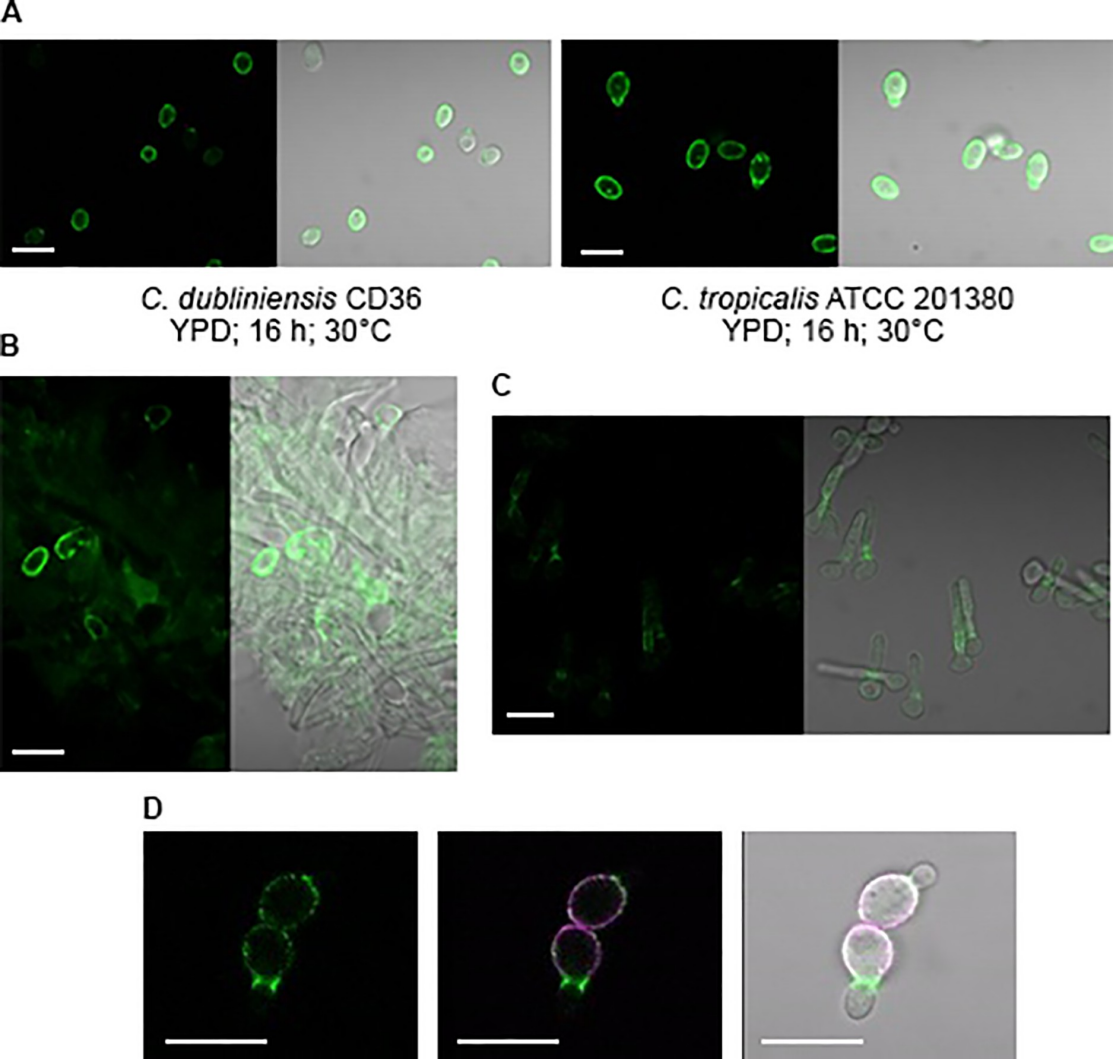
Immunolabeling of *Candida* cells with anti-Als2 MAb 2-C6. (A) *C*. *dubliniensis* CD36 and *C*. *tropicalis* ATCC 201380 yeast cells grown in YPD medium for 16 h at 30°C. Both species were immunolabeled with anti-Als2 2-C6 suggesting a conserved epitope among the species. (B) Anti-Als2 immunolabeling of *C*. *albicans* CAI12 cells recovered from a murine model of oral candidiasis. Some Als2-positive yeast cells were located although they were rare *in vivo*. Als2-positive hyphae were not observed. (C) Various presentations of Als2 localization depending on growth conditions. *C*. *albicans* strain CAI12 was grown in YPD medium for 16 h at 37°C, washed, counted, and resuspended in RPMI medium for 2 h at 30°C. On some germ tubes, Als2 was localized diffusely along the germ tube length while on other cells, Als2 was concentrated at the mother yeast-germ tube junction. (D) *C*. *albicans* strain CAI12 was grown for 16 h in YPD medium at 30°C then labeled directly with Alexa Fluor 594 which appeared as a purple color. Cells were released into fresh YPD medium and grown for 1 h at 30°C and 200 rpm shaking. Als2 localized preferentially to the junction between the mother yeast and bud in newly dividing cells. In all images, the scale bar indicates 10 μm.

MAb 2-C6 immunolabeled the surface of some *C*. *dubliniensis* CD36 cells grown in YPD medium for 16 h at 30°C (Fig 3A). Immunolabeling of *C*. *tropicalis* strain ATCC 201380 was even stronger and more-consistent, suggesting that MAb 2-C6 recognizes cross-reactive epitopes on these species that are closely related to *C*. *albicans*. MAb 2-C7, raised against the Endo H-treated form of Als2, did not cross-react with the *C*. *dubliniensis* or *C*. *tropicalis* strains tested above. MAb 2-C6 immunolabeled *C*. *albicans* yeast cells recovered from a murine model of oral candidiasis (Fig 3B) documenting *ALS2* transcription *in vivo*.

Localization of Als2 was variable among *C*. *albicans* cells from the same culture, as well as influenced by culture conditions. For example, on some germ tubes in Fig 3C, Als2 was displayed diffusely along the germ tube length, while on others, Als2 was focused at the junction between mother cell and germ tube. Alexa Fluor 594 labeling of inoculum cells was used to distinguish them from daughter cells as the culture population expanded. Fig 3D showed that in some yeast cells, Als2 was localized intensely at the mother cell-bud junction and that immunolabeling was more associated with the mother cell than the bud. Additional exploration is required to better understand the general trends noted in this initial study. Availability of the anti-Als2-specific MAbs will facilitate such inquiries.

## Discussion

MAbs specific for *C*. *albicans* Als9-1 (9-C4) and Als9-2 (9-A7) that are capable of distinguishing between the two main variants produced by the *ALS9* locus [13, 14] were raised in mice. Two other MAbs, raised against the glycosylated (2-C6) and Endo H-treated (deglycosylated; 2-C7) form of the Als2 NT domain [7], were also created. While each of the anti-Als2 MAbs are suitably specific for Als2 in ELISA screening, MAb 2-C6 is preferred because of its negative background when immunolabeling *C*. *albicans* strain 2757 (Fig 2). Previous work described specific anti-Als MAbs including 1-B2 (anti-Als1) [8], 3-A5 (anti-Als3) [7], 4-A1 (anti-Als4) [10], 5-A5 (anti-Als5) [7, 9], and 6-A1 (anti-Als6) [7]. All anti-Als hybridomas listed here were deposited in the Developmental Studies Hybridoma Bank (University of Iowa; https://dshb.biology.uiowa.edu) so that they are available to the research community. These new MAbs complete the collection for the Als family in *C*. *albicans*, except Als7.

Attempts to raise a MAb specific for *C*. *albicans* Als7, using the NT domain (amino acids 19–332) produced in *P*. *pastoris* [7] were unsuccessful; none of the resulting antibodies were specific for Als7 when assayed by ELISA. Despite the impressive number of *ALS7* alleles that have been documented, Als7 is predicted to have a minor presence on the *C*. *albicans* surface due to low expression levels of the gene [27]. TaqMan measurement of *ALS7* expression levels suggest they are as low as *ALS6* expression levels in standard *in vitro* culture conditions [28]. In fact, previous work used the anti-Als6 MAb 6-A1 as a negative control in adhesion assays since its interaction with the cell surface could not be visualized by immunofluorescent microscopy [7]. It is expected that an anti-Als7 MAb would require an *ALS7* overexpression strain to visualize surface protein, much like the *ALS9* overexpression strains described here. The need for *ALS9* overexpression strains to visualize Als9 was unanticipated. For example, measurement of *ALS* transcription levels suggested similar results for *ALS2* and *ALS9* in the diploid *C*. *albicans* [28]. It is possible that the haploid dosage of *ALS9-1* and *ALS9-2* essentially halved protein abundance, dropping it below the detection limit for immunolabeling and fluorescent microscopy.

Work with the entire *C*. *albicans* anti-Als MAb collection demonstrated that it is easiest to visualize Als proteins produced from the most-highly-expressed *ALS* genes, confirming a positive association between transcriptional activity and protein abundance across the family. For example, Als3 is readily apparent on the length of *C*. *albicans* germ tubes and hyphae, consistent with strong *ALS3* expression during hypha formation [7]. Als1 covers the surface of yeast cells except bud scars and is localized proximal to the mother yeast as a germ tube emerges [8]. The burst of *ALS1* expression that accompanies release of cells from a saturated culture into fresh growth medium results in strong Als1 production on the first daughter yeast, as well as on some of the granddaughters. Als1 persistence on the surface of these yeast cells during subsequent doublings leads to a heterogeneous anti-Als1 immunolabeling population in a culture flask. A similar heterogeneous cell population was observed here for anti-Als2 immunolabeling (Fig 2) although the kinetics of *ALS2* gene expression and protein production remain to be defined.

Als2 was detectable on the *C*. *albicans* cell surface using anti-Als2 MAb 2-C6 although its distribution varied among individual cells and incubation conditions (Figs 2 and 3). Rare Als2-positive yeast cells were recovered from a murine model of oral candidiasis (Fig 3). Anti-Als immunolabeling of *C*. *albicans* cells *in vivo* were noted with MAbs against some of the other proteins produced from highly expressed *ALS* genes. For example, anti-Als3 immunolabeled *C*. *albicans* hyphae in a formalin-fixed, paraffin-embedded mouse kidney section as well as hyphae dissected from kidney tissue [7]. Als1 and Als4 were visualized on *C*. *albicans* yeast and hyphae dissected from a mouse kidney and fungal cells recovered from a murine model of oral candidiasis [10]. Compared to the limited localization of Als1 and Als4 at the mother yeast-germ tube junction on hyphae *in vitro*, protein was detected over greater lengths of hyphae *in vivo*, suggesting differences in Als protein production *in vivo* compared to a culture flask.

Little is known about Als2, relative to some of the other proteins in the *C*. *albicans* Als family. Fanning et al. [29] demonstrated that *ALS2* (as well as *ALS1* and *ALS4*) is repressed by the catalytic protein kinase A subunit Tpk1: expression of these *ALS* genes increases approximately 5- to 20-fold in a *tpk1/tpk1 C*. *albicans* strain. *ALS2* overexpression makes *C*. *albicans* hypersensitive to caspofungin and results in significantly decreased cell-wall depth compared to wild-type cells. Dissection of the contributions of the Als proteins to adhesion, cell wall integrity, and cell wall structure suggested that Als2 functions in all three capacities.

Previous work indicated difficulty with deleting the second *ALS2* allele to create a null *C*. *albicans* strain [12], a result echoed by Fanning et al. [29] and consistent with an essential role for Als2 in cell wall biogenesis. In the previous study [12], the maltose-inducible *MAL2* promoter was placed upstream of the intact *ALS2* allele in a *als2/ALS2* heterozygous strain. Under non-inducing conditions, the resulting strain had *ALS2* expression of 0.36 ± 0.21 fold-change compared to a wild-type control [12]. Growth in the presence of maltose showed as much as a 21-fold increase in *ALS2* transcription compared to the wild-type strain. *ALS4* expression increased by 3.2-fold in the P*MAL2-ALS2* strain under non-inducing conditions suggesting compensatory regulation between the loci. In the present study, strain 2757 showed considerably decreased *ALS2* expression and a concomitant increase in *ALS4* expression, essentially confirming the previous result. Measurable *ALS2* expression in strain 2757 indicates that it is not zero, so a true *als2/als2* null has yet to be constructed. Despite the compensatory increase in *ALS4* expression, results from Fanning et al. [29] demonstrated that Als4 does not affect caspofungin sensitivity or cell-wall depth so Als2 and Als4 do not fully overlap functionally.

The anti-Als2 MAb 2-C6 was notable because of its cross-reactivity with *C*. *dubliniensis* and *C*. *tropicalis*. Another anti-Als MAb, anti-Als4 4-A1, recognized the surface of *C*. *tropicalis* [10]. Although detailed and accurate sequences for the *ALS* loci are now available for *C*. *albicans*, *C*. *dubliniensis* [30], and *C*. *tropicalis* [28], the information does not shed much light on the basis for cross-reactivity of anti-Als2 2-C6 or anti-Als4 4-A1 with *C*. *dubliniensis* and/or *C*. *tropicalis*. For example, the *C*. *dubliniensis* positional ortholog of *C*. *albicans ALS2* is *Cd64800*, a gene for which an identical copy is present at another locus (*Cd65010*) [25, 30]. TaqMan assays cannot distinguish between these identical loci which produce extremely high expression levels in cells grown under standard *in vitro* culture conditions [28]. Within the immunogen region (amino acids 18–328) [7], *C*. *albicans* Als2 is 71% identical to the *C*. *dubliniensis* proteins. The *C*. *albicans* Als2 immunogen is also 74% identical to Cd64210, the protein predicted from the *ALS1* positional ortholog *Cd64210* which is also expressed at a level that would promote immunolabeling detection of the encoded protein [28]. It is unclear whether either of these proteins is responsible for the cross-reactivity of anti-Als2 MAb 2-C6.

In *C*. *tropicalis*, *CtrALS3786* is the positional ortholog of *C*. *albicans ALS2* [25]. However, *CtrALS3786* expression levels are not particularly high and the two proteins share only 50% identity [2, 28]. *C*. *albicans ALS4* does not have a predicted positional ortholog in the *C*. *tropicalis* genome and its protein immunogen sequence is only approximately 50% identical to the same region of the *C*. *tropicalis* proteins. Several of the *C*. *tropicalis ALS* genes are highly expressed under typical *in vitro* culture conditions (e.g. *CtrALS2293*, *CtrALS3882-1*, *CtrALS1028*) and may produce the protein that responsible for the cross-reactive immunolabeling signals [28].

Much remains to be learned about Als2 and other proteins in the *C*. *albicans* Als family. The availability of MAbs to specifically recognize individual proteins, and even proteins produced by allelic variants such as *ALS9-1* and *ALS9-2*, will aid future investigations into Als protein function and diversity of the Als family.

## Supporting information

S1 Raw images Original gel and blot images for Figs 1A and 2A. (PDF)
