# Supplementary material for: Development and validation of monoclonal antibodies specific for Candida albicans Als2, Als9-1, and Als9-2
Source: PLoS One. 2022 Jul 8;17(7):e0269681. doi: 10.1371/journal.pone.0269681 (PMC9269773; doi:10.1371/journal.pone.0269681)
Supplement: S1 Raw images — (PDF) [file pone.0269681.s001.pdf]

S1 Fig. Original Gel and Blot Images for Fig 1A and Fig 2A.

Original gel and blot images for Fig 1A

9L 9S

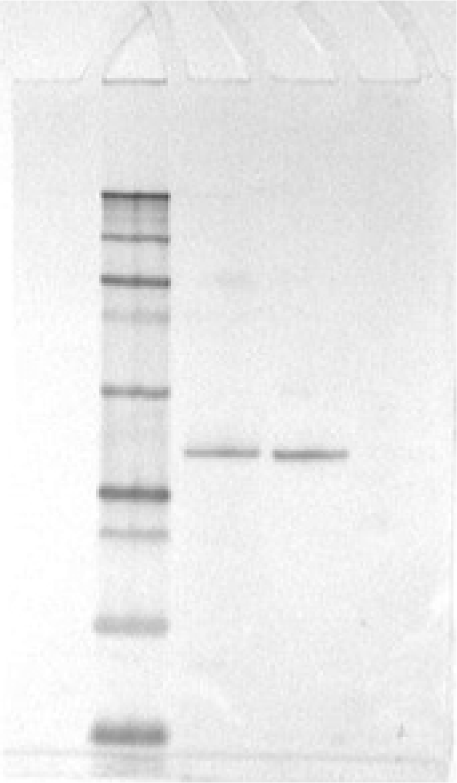

Silver Stain

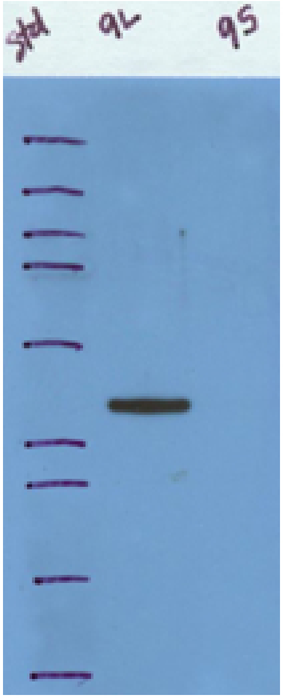

Anti-Als9-1
(called 9L)

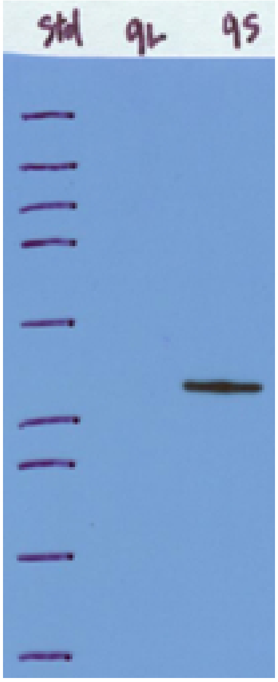

Anti-Als9-2
(called 9S)

Original Gel and Blot Images for Fig 2A.

Als2G
Als2De
Als3
Als4

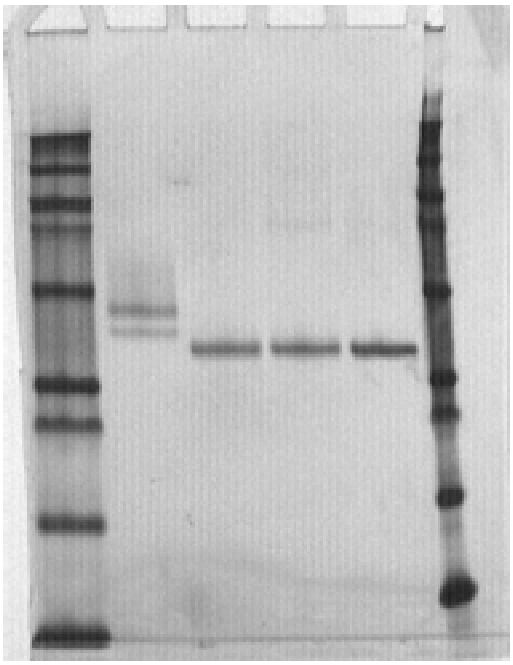

Silver Stain

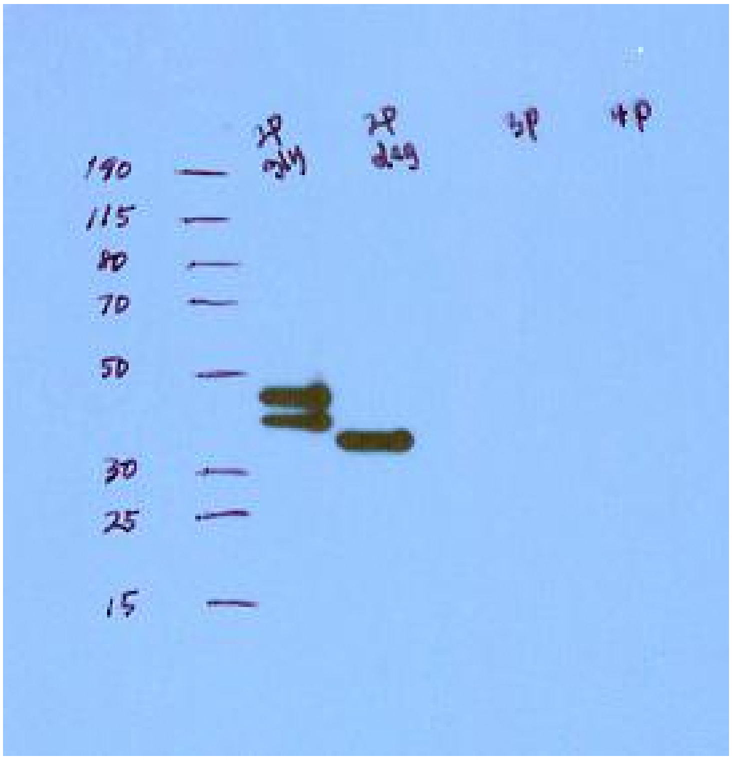

Anti-Als2G
